# Assessment of Gaps in Knowledge, Attitudes, and Practices Regarding Antenatal Care Among Primigravida Women in the First Trimester of Pregnancy and Their Information-Seeking Behavior

**DOI:** 10.7759/cureus.112670

**Published:** 2026-07-14

**Authors:** Bindiya Gupta, Kanchan Bala, Sanchita Pugazhendi, Kamli Prakash

**Affiliations:** 1 Obstetrics and Gynecological Nursing, Himalayan College of Nursing, Swami Rama Himalayan University, Dehradun, IND; 2 Community Health Nursing, Himalayan College of Nursing, Swami Rama Himalayan University, Dehradun, IND; 3 Medical Surgical Nursing, Himalayan College of Nursing, Swami Rama Himalayan University, Dehradun, IND

**Keywords:** antenatal care, attitude, india, knowledge, practice, pregnancy, primigravida, uttar pradesh

## Abstract

Background

Good quality antenatal care (ANC) lays the foundation for a healthy pregnancy and favorable maternal and neonatal outcomes. Low utilization of ANC services is a global public health problem leading to preventable maternal and neonatal mortality and morbidity. This study aimed to assess the knowledge, attitudes, and practices (KAP) of primigravida women regarding ANC in the first trimester of pregnancy and to examine their associations with demographic variables at a selected community health center (CHC) in the district of Gautam Buddha Nagar, Uttar Pradesh. It also identified preferred ANC information topics and information sources among primigravida women to highlight gaps and support the development of targeted antenatal educational interventions to improve utilization of ANC services.

Materials and methods

The study employed a quantitative descriptive design using first-trimester pre-intervention baseline data from 124 primigravida women enrolled in a randomized controlled trial. One CHC in the Gautam Buddha Nagar district, Uttar Pradesh, was selected by lottery, and participants were recruited from it using a non-probability consecutive sampling technique. Data were collected using a demographic tool, a structured knowledge questionnaire, a five-point Likert attitude scale, and a self-reported practice checklist and were analyzed using SPSS Statistics version 20.0 (IBM Corp. Released 2011. IBM SPSS Statistics for Windows, Version 20.0. Armonk, NY: IBM Corp.).

Results

The majority of participants demonstrated average knowledge (87, 70.2%) and good practices (103, 83.1%), while 64 (51.6%) showed a positive attitude toward ANC in the first trimester of pregnancy. The mean KAP scores were 26 ± 6.3, 111.8 ± 9.6, and 8.8 ± 1.4, respectively. Significant positive correlations existed between knowledge and attitude (0.59), attitude and practice (0.36), and knowledge and practice (0.38) at p < 0.001. Age, education, place of residence, and residence type were significantly associated with ANC knowledge (p < 0.05). Education, monthly family income, residence type, and mode of transport used to reach the CHC were significantly associated with attitudes toward ANC (p < 0.05). Family members and friends were the main sources of ANC information. Nutrition and diet were the most preferred topic of information.

Conclusions

The majority of primigravida women had average knowledge and good practices, and nearly half had a positive attitude toward ANC in the first trimester of pregnancy. The study findings highlight the need for targeted antenatal educational interventions that involve family members to further enhance their knowledge and attitudes toward ANC, thereby contributing to better utilization of ANC services and improved maternal and fetal health.

## Introduction

Pregnancy is a developmental transition during which physical and physiological changes take place in the body of a woman to accommodate the growing fetus. Despite the global efforts to strengthen the healthcare delivery system and improve accessibility, many women die due to preventable pregnancy- and childbirth-related causes. According to the World Health Organization (WHO), nearly 0.26 million women died following pregnancy and childbirth in 2023, and almost two million babies are stillborn every year [1,2]. Additionally, approximately 13.4 million premature births and nearly 19.8 million low birth weight babies were reported globally in 2020 [3,4]. To reduce preventable mortality and morbidity and promote health and well-being, WHO recommends eight antenatal care (ANC) contacts during pregnancy, with the first occurring within the first 12 weeks of gestation [5]. According to the United Nations Children's Fund (UNICEF) global databases (2024), 87% of pregnant women attended at least one ANC visit, while 70% had four or more ANC visits worldwide. In Western and Central Africa and South Asia, which have the highest maternal deaths, 57% and 58% of pregnant women, respectively, had four or more ANC visits [6]. Despite the proven benefits of early ANC for maternal and fetal health, evidence from Africa, India, and Uttar Pradesh suggests that many women do not seek ANC during the first trimester of pregnancy [7-9]. This may represent a missed opportunity for timely folic acid supplementation, essential investigations, early risk screening, health education, and care [9]. Inadequate ANC is one of the major risk factors for maternal death in India [10]. It is often observed among poor, less-educated, and less-empowered women residing in rural areas [11].

Good ANC during pregnancy and optimal utilization of ANC services depend largely on women’s ANC knowledge, its purpose, the right time to initiate it, perceived benefits, the quality of ANC services available to them, and awareness of their own health needs during pregnancy [12-15]. Many contextual factors also affect ANC utilization [16] and, in turn, maternal and neonatal outcomes. Pregnant women and their families have frequently expressed dissatisfaction with antenatal counseling services due to the gap between expectation and information delivery [15]. Evidence from low- and middle-income countries has also revealed inadequate quality and content of ANC services, highlighting important gaps in service delivery [17]. These inadequacies can negatively impact pregnant women’s ANC-related knowledge, attitudes, and practices (KAP) and discourage optimal utilization of ANC services. In this context, ANC services represent a critical area for improvement.

Gravidity is an important determinant of ANC knowledge. Primigravida women are particularly vulnerable to inadequate ANC due to lack of prior pregnancy experience. They may have limited knowledge of ANC and danger signs, as well as low awareness of ANC services available to them. Poor knowledge can lead to inadequate practices and poor ANC service utilization, thereby increasing the risk of maternal and neonatal complications [9,14]. Initiation of ANC visits during the first trimester has been associated with a reduced risk of low birth weight [9]. However, primigravida women’s KAP regarding ANC may influence the timely and optimal utilization of ANC services. It is therefore pertinent to analyze gaps in ANC-related KAP, as well as ANC information sources and priorities among primigravida women in the first trimester of pregnancy, to design effective antenatal educational interventions and optimize their utilization of ANC services. Examining the association between demographic variables and KAP can help identify women at greater risk of poor ANC. Overall, the findings are expected to support the development of women-centric, evidence-based, context-specific, and targeted interventions with greater acceptability among end users. This may also contribute to the formulation of equitable maternal health policies and programs. Such efforts would ultimately contribute to enhanced ANC service utilization and reduction in avoidable maternal and neonatal morbidity and mortality while promoting positive childbirth experiences.

The present study aimed to assess the KAP of primigravida women regarding ANC in the first trimester of pregnancy and to determine the associations of these variables with demographic characteristics at a community health center (CHC) in Gautam Buddha Nagar, Uttar Pradesh. It also aimed to determine participants' preferences for ANC information and sources.

## Materials and methods

Study design and setting

This study employed a quantitative descriptive design using first-trimester pre-intervention baseline data of all 124 participants enrolled in a randomized controlled trial (RCT). It was conducted at a CHC in the Gautam Buddha Nagar district of Uttar Pradesh, selected by lottery. Baseline data collection was done in November 2024.

Study population and sampling

First-trimester primigravida women were recruited from the obstetrics and gynecology outpatient department of the selected CHC for the parent study. Non-probability consecutive sampling was used to recruit participants until the required sample size was met. The first trimester was confirmed through antenatal records or ultrasound reports. Participants were screened based on additional inclusion criteria, including singleton pregnancy (confirmed by ultrasound), self-reported desire to continue the pregnancy, CHC registration, and willingness to provide written informed consent. Women with a history of chronic medical or psychiatric illness identified through self-report or antenatal/medical records were excluded. Women unwilling to take part in the RCT were also excluded. Of 175 primigravida women assessed for eligibility, 49 were excluded for not meeting the inclusion criteria, and two refused to participate, leaving a final sample size of 124.

Ethical clearance was given by the Ethics Committee of Swami Rama Himalayan University (approval number: SRHU/HIMS/E-1/2023/83), the Dehradun Ethics Committee, and administrative permission was granted by the Chief Medical Officer of Gautam Buddha Nagar district. Written informed consent was obtained from all participants before data collection, after informing them about the study and ensuring that confidentiality, autonomy, and the voluntary nature of participation were upheld.

Data collection tools

Data were collected through face-to-face interviews using a demographic tool, a structured knowledge questionnaire, a five-point Likert attitude scale, and a self-reported practice checklist. Each KAP tool had four sections to assess KAP of primigravida women across all three trimesters of pregnancy. In the first trimester (average gestational age of 11.4 weeks), data were collected using section A of the KAP tools, which included items on ANC visits and assessments, government maternal and child health (GMCH) schemes, respectful maternity care (RMC), and minor ailments of pregnancy. The demographic tool included participants’ information on their age, religion, education of self and husband, occupation, monthly family income, type of family, place of living, residence type, distance from CHC, mode of transport used for CHC (private vehicles or others: walking/public transport), previous ANC knowledge with their source of ANC information (family and friends, healthcare workers/services, social media, books, or antenatal classes), and desire to receive antenatal education with preferred topics.

The knowledge questionnaire section contained 29 multiple-choice questions. Questions on ANC visits and assessments included the recommended number and purpose of ANC visits; the role of folic acid; investigations performed during pregnancy and their importance; and the purpose and schedule of the tetanus and diphtheria vaccine. Questions on GMCH schemes included benefits and service-providing institutions, financial assistance schemes, and money transfer. RMC-related questions included examples of disrespectful maternity care and RMC. Questions on minor ailments covered management of morning sickness, acid reflux, constipation, leg swelling, varicose veins, frequent micturition, correct posture during sitting, lifting and lying down, identification of danger signs of pregnancy (vaginal bleeding, extreme swelling of hands or face, severe nausea and vomiting, foul-smelling vaginal discharge, blurred vision, etc.), and consultation for medication. Each correct response was awarded one mark. Questions with multiple correct responses were awarded 1 mark per correct response, resulting in a total knowledge score of 44. ANC knowledge level was classified as poor (score: 0-14), average (score: 15-29), and good (score: 30-44).

The attitude scale section contained 28 statements. It assessed participants’ opinions regarding the number of ANC visits, first-trimester ANC attendance and investigations, home delivery, GMCH scheme registration and adequacy, disrespectful care acceptance and its effect, use of home remedies and healthcare provider consultation for minor ailments, effect of dietary modification and posture on minor ailments, folic acid role, and medicine safety and danger signs requiring hospital visits during pregnancy. The total attitude score ranged from 28 to 140. Positive items (19) were scored from 1 (strongly disagree) to 5 (strongly agree), and negative items (9) were scored in a reverse order of 5 (strongly disagree) to 1 (strongly agree). Attitude toward the ANC was classified as negative (scores: 28-84), neutral (scores: 85-112), and positive (scores: 113-140).

The practice checklist section contained 11 dichotomous (yes/no) items. It inquired about first-trimester ANC attendance, regular weight monitoring, registration under GMCH schemes, and dietary and lifestyle practices, such as avoiding foods that cause vomiting, tea or coffee consumption, lying down immediately after eating, prolonged standing, and delaying urination. It also asked about folic acid intake and healthcare provider consultation before using medicine or a home remedy for minor ailments like nausea and vomiting, constipation, acid reflux, etc. Each correct answer was awarded one mark. Total practice score ranged from 0 to 11. ANC practice level was classified as poor (scores: 0-3), average (scores: 4-7), and good (scores: 8-11).

Tools were validated by eight subject-matter experts from the nursing field and the medical specialties of obstetrics and gynecology, as well as community medicine. All three tools, i.e., structured knowledge questionnaire (Cronbach’s alpha = 0.89), attitude scale (Karl Pearson coefficient = 0.97), and self-reported practice checklist (Karl Pearson coefficient = 0.98), showed good reliability.

Data analysis

Data were analyzed using SPSS Statistics version 20.0 (IBM Corp. Released 2011. IBM SPSS Statistics for Windows, Version 20.0. Armonk, NY: IBM Corp.). Frequencies and percentages were used to summarize categorical variables. Mean, SD, and mean percentage were used for continuous variables. The chi-square test was used to assess the statistical significance of the association between demographic variables and KAP scores at a significance level of p < 0.05. Pearson correlation between KAP variables was considered significant at p < 0.05. Bar charts were used to visually represent the data.

## Results

Demographic characteristics

A total of 124 primigravida women participated in the study. Seventy-eight (62.9%) were in the 20-25-year age group. The majority were Hindus (111, 89.5%). Most participants (45, 36.3%) and most husbands (52, 41.9%) had secondary to higher secondary level education. One hundred and fifteen (92.7%) were homemakers, and 63 (50.8%) had a monthly family income of INR 10,000 to INR 22,000. Eighty-five (68.5%) lived in joint families. The majority came from semi-rural areas (99, 79.8%). 62 (50.0%) lived in rented houses, 85 (68.5%) lived within 10 km of the selected CHC, and 78 (62.9%) didn’t use private transport to reach the CHC. Sixty-seven (54.0%) had no previous ANC knowledge, and the majority desired antenatal education (112, 90.3%) (Table 1).

**Table 1:** Demographic characteristics of the study participants (N = 124) INR: Indian rupee, CHC: community health center, ANC: antenatal care

Demographic variables	Frequency (%)
Age	
19 years	21 (16.9)
20-25 years	78 (62.9)
26-35 years	25 (20.2)
Religion	
Hindu	111 (89.5)
Muslim	13 (10.5)
Education	
No formal education	14 (11.3)
Primary to middle	26 (20.9)
Secondary to higher secondary	45 (36.3)
Graduate and above	39 (31.5)
Occupation	
Homemaker	115 (92.7)
Working	9 (7.3)
Education (husband)	
No formal education	12 (9.7)
Primary to middle	18 (14.5)
Secondary to higher secondary	52 (41.9)
Graduate and above	42 (33.9)
Monthly family income (INR)	
10,000-22,000	63 (50.8)
22,001-34,000	31 (25.0)
34,001-46,000	14 (11.3)
46,001-65,000	16 (12.9)
Family type	
Joint family	85 (68.5)
Nuclear family	39 (31.5)
Place of living	
Urban	25 (20.2)
Semi-rural	99 (79.8)
Residence type	
Permanent	62 (50.0)
Rented	62 (50.0)
Distance from CHC	
0-10 km	85 (68.5)
>10-17 km	39 (31.5)
Mode of transport to CHC	
Others	78 (62.9)
Private	46 (37.1)
Previous ANC knowledge	
Yes	57 (46.0)
No	67 (54.0)
ANC education desired	
Yes	112 (90.3)
No	12 (9.7)

KAP regarding ANC

The majority of participants demonstrated average knowledge (87, 70.2%) and good practices (103, 83.1%) toward ANC in the first trimester of pregnancy. Nearly half had a positive attitude toward ANC (64, 51.6%) (Table 2).

**Table 2:** Distribution of the study participants according to their KAP levels regarding ANC (N = 124) ANC: antenatal care, KAP: knowledge, attitudes, and practices

Variable	KAP level regarding ANC		
Knowledge	Good	Average	Poor
	32 (25.8)	87 (70.2)	5 (4.0)
Attitude	Positive	Neutral	Negative
	64 (51.6)	60 (48.4)	0
Practice	Good	Average	Poor
	103 (83.1)	21 (16.9)	0

The mean knowledge score was 26 ± 6.3, the mean attitude score was 111.8 ± 9.6, and the mean practice score was 8.8 ± 1.4 (Table 3).

**Table 3:** Mean, SD, and mean percentage of KAP scores regarding ANC among the study participants during the first trimester (N = 124) ANC: antenatal care, KAP: knowledge, attitudes, and practices, SD: standard deviation

Variable	Maximum score	Mean ± SD	Mean %
Knowledge	44	26 ± 6.3	59.0
Attitude	140	111.8 ± 9.6	79.8
Practice	11	8.8 ± 1.4	80.0

Significant positive correlations were observed between knowledge and attitude (r = 0.59, p < 0.001), attitude and practice (r = 0.36, p < 0.001), and knowledge and practice (r = 0.38, p < 0.001) (Table 4).

**Table 4:** Correlation between the KAP scores regarding ANC among the study participants (N = 124) * significant at p < 0.05. KAP: knowledge, attitudes, and practices

Variables	Pearson correlation coefficient	p-value
Knowledge and attitude	0.59	<0.001*
Attitude and practice	0.36	<0.001*
Knowledge and practice	0.38	<0.001*

Association between KAP regarding ANC and demographic variables

A statistically significant association was observed between participants' first-trimester ANC knowledge score and their age (p < 0.001), education (p = 0.024), place of residence (p < 0.001), and residence type (p = 0.031) (Table 5).

**Table 5:** Association between first-trimester ANC knowledge scores and the demographic characteristics of the study participants (N = 124) * significant at p < 0.05, ^$ ^continuity correction is used INR: Indian rupee, CHC: community health center, ANC: antenatal care

Demographic variables	First-trimester knowledge score (median score = 27)			
	At and above median	Below median	χ²	p-value
	Frequency (%)	Frequency (%)		
Age in years				
19-26	44 (43.1)	58 (56.9)	13.529	<0.001*
27-35	19 (86.4)	3 (13.6)		
Religion				
Hindu	55 (49.5)	56 (50.5)	0.669	0.56
Muslim	8 (61.5)	5 (38.5)		
Education				
No formal education	3 (21.4)	11 (78.6)	5.45	0.024*
Educated	60 (54.5)	50 (45.5)		
Occupation				
Homemaker	57 (49.6)	58 (50.4)	0.412	0.521^$^
Working	6 (66.7)	3 (33.3)		
Education (husband)				
No formal education	3 (25.0)	9 (75.0)	3.54	0.073
Educated	60 (53.6)	52 (46.4)		
Monthly family income (INR)				
10,000-22,000	30 (47.6)	33 (52.4)	0.521	0.479
22,001-65,000	33 (54.1)	28 (45.9)		
Family type				
Joint	43 (50.6)	42 (49.4)	0.005	1
Nuclear	20 (51.3)	19 (48.7)		
Place of living				
Semi-rural	41 (41.4)	58 (58.6)	17.331	<0.001*
Urban	22 (88.0)	3 (12.0)		
Residence type				
Permanent	38 (61.3)	24 (38.7)	5.453	0.031*
Rented	25 (40.3)	37 (59.7)		
Distance from CHC				
0-10 km	42 (49.4)	43 (50.6)	0.21	0.701
>10-17 km	21 (53.8)	18 (46.2)		
Mode of transport to CHC				
Others	35 (44.9)	43 (55.1)	2.963	0.097
Private	28 (60.9)	18 (39.1)		
Previous ANC knowledge				
Yes	33 (57.9)	24 (42.1)	2.121	0.155
No	30 (44.8)	37 (55.2)		

The first-trimester attitude score of participants toward ANC was significantly associated with their education (p = 0.021), monthly family income (p = 0.013), residence type (p < 0.001), and mode of transport used to reach the CHC (p = 0.04) (Table 6).

**Table 6:** Association between first-trimester ANC attitude scores and the demographic characteristics of the study participants (N = 124) * significant at p < 0.05, ^$ ^continuity correction is used INR: Indian rupee, CHC: community health center, ANC: antenatal care

Demographic variables	First-trimester attitude score (median score = 113)			
	At and above median	Below median	χ²	p-value
	Frequency (%)	Frequency (%)		
Age in years				
19-26	51 (50.0)	51 (50.0)	1.349	0.347
27-35	14 (63.6)	8 (36.4)		
Religion				
Hindu	58 (52.3)	53 (47.7)	0.012	1
Muslim	7 (53.8)	6 (46.2)		
Education				
No formal education	3 (21.4)	11 (78.6)	6.077	0.021*
Educated	62 (56.4)	48 (43.6)		
Occupation				
Homemaker	60 (52.2)	55 (47.8)	0	1.000^$^
Working	5 (55.6)	4 (44.4)		
Education (husband)				
No formal education	5 (41.7)	7 (58.3)	0.616	0.547
Educated	60 (53.6)	52 (46.4)		
Monthly family income (INR)				
10,000-22,000	26 (41.3)	37 (58.7)	6.383	0.013*
22,001-65,000	39 (63.9)	22 (36.1)		
Family type				
Joint	47 (55.3)	38 (44.7)	0.895	0.439
Nuclear	18 (46.2)	21 (53.8)		
Place of living				
Semi-rural	49 (49.5)	50 (50.5)	1.684	0.263
Urban	16 (64.0)	9 (36.0)		
Residence type				
Permanent	44 (71.0)	18 (29.0)	17.11	<0.001*
Rented	21 (33.9)	41 (66.1)		
Distance from CHC				
0-10 km	47 (55.3)	38 (44.7)	0.895	0.439
>10-17 km	18 (46.2)	21 (53.8)		
Mode of transport to CHC				
Others	35 (44.9)	43 (55.1)	4.802	0.04*
Private	30 (65.2)	16 (34.8)		
Previous ANC knowledge				
Yes	34 (59.6)	23 (40.4)	2.211	0.152
No	31 (46.3)	36 (53.7)		

No significant association was found between the first-trimester ANC practice score of the participants and their demographic variables (p < 0.05) (Table 7).

**Table 7:** Association between first-trimester ANC practice scores and the demographic characteristics of the study participants (N = 124) * significant at p < 0.05, ^$ ^continuity correction is used INR: Indian rupee, CHC: community health center, ANC: antenatal care

Demographic variables	First-trimester practice score (median score = 9)			
	At and above median	Below median	χ²	p-value
	Frequency (%)	Frequency (%)		
Age in years				
19-26	61 (59.8)	41 (40.2)	0.54	0.485
27-35	15 (68.2)	7 (31.8)		
Religion				
Hindu	69 (62.2)	42 (37.8)	0.34	0.765
Muslim	7 (53.8)	6 (46.2)		
Education				
No formal education	8 (57.1)	6 (42.9)	0.11	0.776
Educated	68 (61.8)	42 (38.2)		
Occupation				
Homemaker	69 (60.0)	46 (40.0)	0.49	0.484^$^
Working	7 (77.8)	2 (22.2)		
Education (husband)				
No formal education	6 (50.0)	6 (50.0)	0.28	0.594^$^
Educated	70 (62.5)	42 (37.5)		
Monthly family income (INR)				
10,000-22,000	40 (63.5)	23 (36.5)	0.26	0.713
22,001-65,000	36 (59.0)	25 (41.0)		
Family type				
Joint	51 (60.0)	34 (40.0)	0.19	0.696
Nuclear	25 (64.1)	14 (35.9)		
Place of living				
Semi-rural	58 (58.6)	41 (41.4)	1.51	0.257
Urban	18 (72.0)	7 (28.0)		
Residence type				
Permanent	37 (59.7)	25 (40.3)	0.14	0.854
Rented	39 (62.9)	23 (37.1)		
Distance from CHC				
0-10 km	49 (57.6)	36 (42.4)	1.51	0.24
>10-17 km	27 (69.2)	12 (30.8)		
Mode of transport to CHC				
Others	45 (57.7)	33 (42.3)	1.15	0.342
Private	31 (67.4)	15 (32.6)		
Previous ANC knowledge				
Yes	38 (66.7)	19 (33.3)	1.29	0.273
No	38 (56.7)	29 (43.3)		

Information-seeking behavior

**Figure 1:**
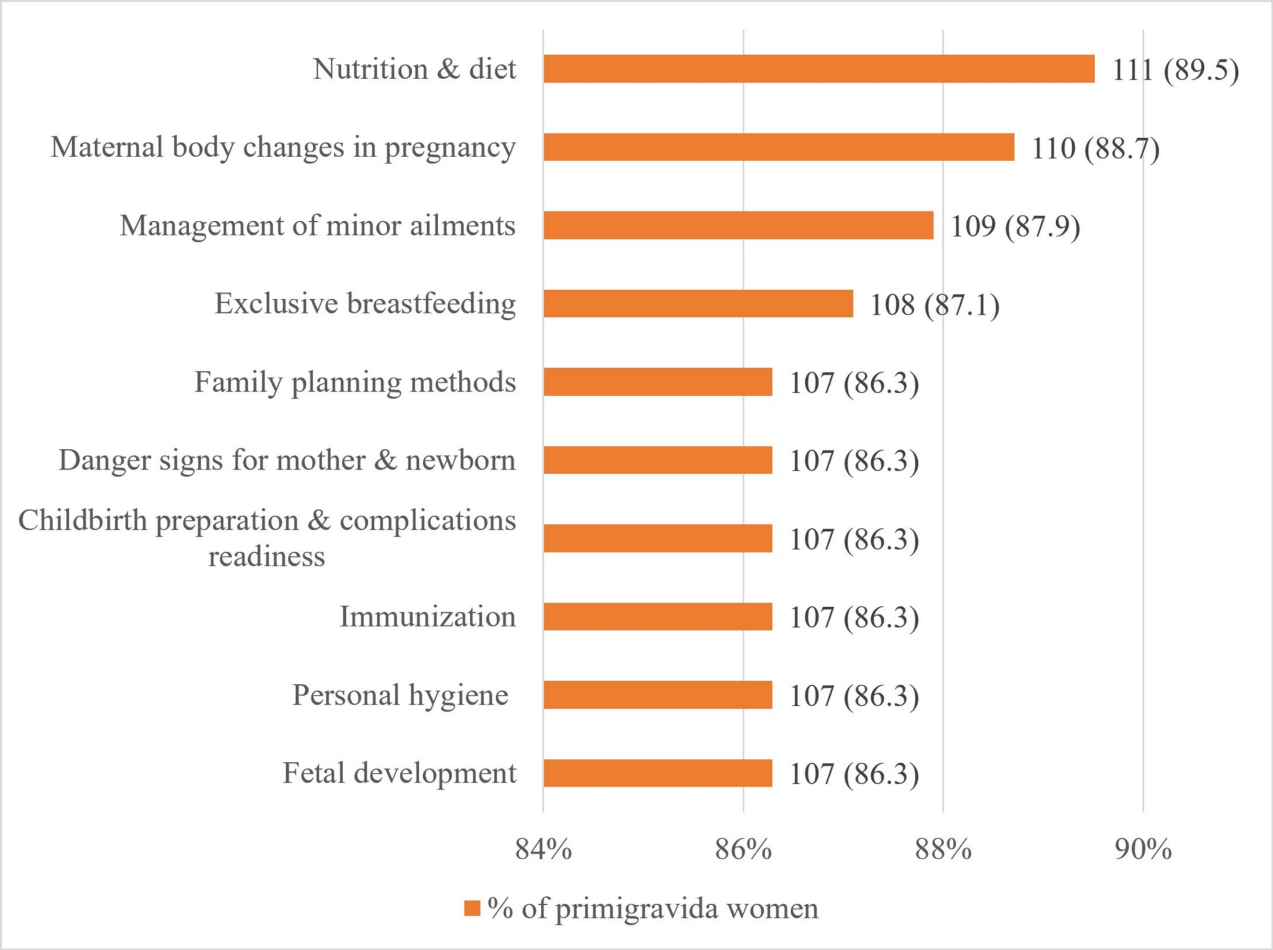
Distribution of the study participants according to their preferred ANC information topics (N = 124) Participants could select more than one topic. ANC: antenatal care

The majority of the participants (111, 89.5%) wanted information on nutrition and diet, followed by changes in the maternal body during pregnancy (110, 88.7%) and the management of minor ailments (109, 87.9%) (Figure 1).

**Figure 2:**
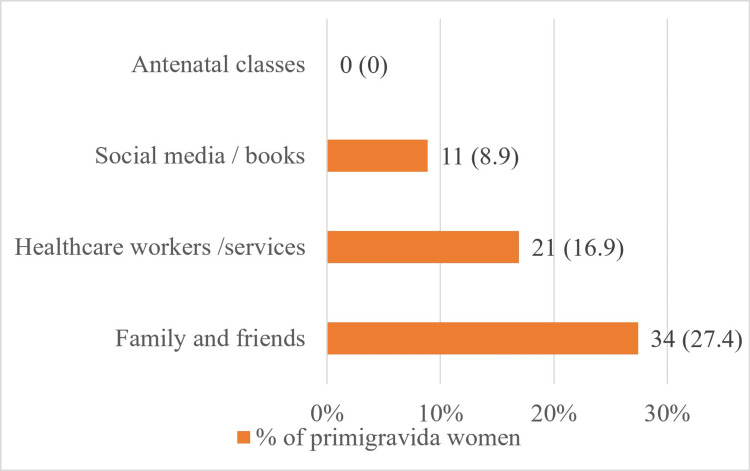
Distribution of the study participants according to their ANC information sources (N = 124) Participants could select more than one source. ANC: antenatal care

Inner circle members, such as family and friends, emerged as the predominant source of ANC information, reported by 34 (27.4%) participants, followed by healthcare workers (21, 16.9%), mostly ASHA workers. Only 11 (8.9%) reported use of social media or books (Figure 2).

## Discussion

This study found an average level of knowledge (87, 70.2%), positive attitude (64, 51.6%), and good practices (103, 83.1%) related to ANC among primigravida women in the first trimester of pregnancy. Bashir et al. reported similar findings of average knowledge (96.0%), positive attitude (98.75%), and good practices (58.5%) toward ANC among 400 pregnant women [18]. In contrast, Gebremariam et al. reported good knowledge (84.1%) but low practice (45%) regarding ANC among 289 pregnant women [19]. Another study by Elmi et al. also reported good ANC knowledge (73%) among 384 pregnant women [20]. Both studies reported a high level of attitude among the majority of their participants (99% and 75.8%, respectively), similar to our findings [19,20]. Observed disparities in KAP across studies can be attributed to differences in demographic and cultural characteristics, variations in the gravidity or parity of study participants, and differences in the tools and cutoff criteria used to assess KAP [18,21]. Regional variations in the quality and delivery of health care services may also affect participants' KAP levels [22]. The present study's findings underscore the need for targeted awareness and educational interventions to improve primigravida women's ANC knowledge, increase their awareness of its benefits, and foster a positive attitude toward timely and appropriate ANC utilization. Although the majority of primigravida women demonstrated good ANC practices, these initiatives may further reinforce and sustain recommended practices.

The study also revealed significant positive correlations among knowledge and attitude (0.59), attitude and practice (0.36), and knowledge and practice (0.38) at p < 0.001. Our findings align with Bashir et al., who reported a significant positive correlation between knowledge and practice at p < 0.05, and with Elmi et al., who reported significant positive correlations between KAP at p < 0.01 [18,20]. These findings suggest that enhancing ANC knowledge through structured educational interventions during the first trimester of pregnancy may help improve attitudes toward ANC and promote adoption of good ANC practices.

In the present study, formal education was significantly associated with higher knowledge and attitude scores of participants compared to no formal education. This may be explained by the fact that education enhances the ability to seek and understand health-related information, thereby increasing knowledge and promoting a positive attitude toward ANC and related services. Higher age, urban residence, and living in a permanent house were also significantly associated with higher knowledge scores in our study. Previous studies have similarly reported that higher age [19,23], higher education [13,23], and urban residence [24] were significantly associated with higher ANC knowledge; in contrast, Rustagi et al. reported similar ANC knowledge among rural and urban participants [25]. Our study findings highlight disparities in knowledge among younger women and those from less advantaged socioeconomic backgrounds. Initiatives to improve education level and ANC knowledge of such women may enhance their health literacy and promote better utilization of ANC services.

Evidence on the influence of demographic factors on attitudes toward ANC is limited. Higher family income, living in a permanent house, and using a private mode of transport to the CHC were also significantly associated with higher attitude scores in our study, indicating that socioeconomic advantage may contribute to more favorable attitudes toward ANC through improved access to ANC services and supportive resources. Bashir et al. reported a significant association between education and attitude toward ANC assessments in line with our finding [18]. Gebremariam et al. reported no such association, possibly because almost all participants demonstrated uniformly good attitudes toward ANC [19].

Our study found no significant association between ANC practices and demographic variables. Similar findings were reported by Sherif et al., who found no significant association between self-care practices during pregnancy and age, education, or family income of participants, while higher knowledge and favorable attitude were significantly associated with good practices [26]. In contrast, Patel et al. reported a significant association between participants' socioeconomic status and their overall ANC practices [23]. These disparities may be attributed to differences in participant characteristics, study settings, and measurement of ANC practices.

Measures to improve KAP among pregnant women regarding ANC should be designed and implemented with observed demographic influences in mind to contextualize interventions and educational programs. These factors can significantly influence implementation outcomes.

Most women in the present study wanted to gain information about nutrition and diet (111, 89.5%), consistent with findings from earlier studies [27,28]. Pregnant women seek nutrition-related information because maternal nutrition directly affects fetal growth. Women are conscious of pregnancy weight gain, and the majority face digestion-related issues, plus there are food taboos that place undue restrictions on food choices. In contrast, Ghiasi revealed that information about fetal growth and care was the most sought-after topic among pregnant women [29]. The stage of pregnancy and parity may affect pregnant women's information needs. Understanding changing information needs throughout pregnancy and across different parity levels can help provide targeted, need-based information.

Thirty-four (27.4%) of the study participants depended on family members and friends for ANC information. A similar pattern was reported by Appiah et al. [27]. In contrast, other studies identified health professionals [29] or the internet [30] as the primary sources of ANC information. The disparity can be attributed to differences in sociodemographic variables, digital skills, accessibility, perceived trustworthiness of source, parity, etc. [28,29]. Our findings highlight the important role of family members in shaping ANC-related knowledge, practices, and decision-making among primigravida women, while also raising concerns about the quality of information obtained from them. Therefore, awareness and educational programs should also focus on involving family members and improving their ANC-related knowledge and attitudes, alongside those of primigravida women. Guiding pregnant women and their families to reliable, user-friendly online resources can help to avoid misinformation.

Limitations

The study generated valuable insights for researchers and policymakers regarding ANC among first-trimester primigravida women, a clinically important group for early ANC interventions. However, a few limitations should be acknowledged. The use of a single CHC as the research setting and a small sample size may limit the broader generalization of the findings to other populations and healthcare settings. As the present study analyzed baseline data from participants enrolled in an RCT using non-probability consecutive sampling, selection bias cannot be ruled out, further limiting the generalizability of the findings. The cross-sectional nature of the study did not allow for causal inferences. The use of a self-reported practice tool may have introduced recall bias and socially desirable responses.

## Conclusions

Most primigravida women had average ANC knowledge in the first trimester of pregnancy. Nearly half of them showed a positive attitude, while the majority demonstrated good practices toward ANC. Targeted antenatal educational interventions need to be designed and implemented with careful consideration of demographic factors to further enhance knowledge, health literacy, and attitudes toward ANC. Family members should also be actively included in such initiatives, as they are an important source of ANC information and can influence primigravida women’s KAP regarding ANC. This could contribute to improved ANC utilization, improved maternal and fetal health, and the achievement of Sustainable Development Goal 3 targets.
